# Cardiogenic Pneumonia: Unilateral Pulmonary Edema Secondary to Severe Eccentric Mitral Regurgitation

**DOI:** 10.7759/cureus.38894

**Published:** 2023-05-11

**Authors:** Jahangir Rouzbehani Selakhor, Guarina Molina, Nichole Brunton, Manush Ohanyan

**Affiliations:** 1 Internal Medicine, Danbury Hospital/Yale University School of Medicine, Danbury, USA; 2 Vascular Medicine, Mayo Clinic, Rochester, USA

**Keywords:** mitraclip, valve repair, valve replacement, mitral regurgitation, pulmonary edema

## Abstract

Mitral regurgitation (MR), whether primary or secondary, stems from functional or anatomical impairment of components of the mitral apparatus resulting in abnormal blood flow to the left atrium during systole. A common complication is bilateral pulmonary edema (PE), which, in rare instances, may be unilateral and easily misdiagnosed. This case presents an elderly male with unilateral lung infiltrates and progressive exertional dyspnea with the failed treatment of pneumonia. Additional workup, including a transesophageal echocardiogram (TEE), showed severe eccentric MR. He underwent mitral valve (MV) replacement with significant improvement in symptoms.

## Introduction

Cardiogenic pulmonary edema (CPE) results from a disturbance in the Starling forces, more specifically, an increase in the pulmonary capillary pressure. While bilateral PE occurs in most cases, unilateral PE has been described in approximately 2% of chronic mitral regurgitation (MR) cases [1]. The physiology is explained by the anatomy of the mitral valve. As the regurgitant jet is directed toward the right pulmonary veins (particularly the right upper pulmonary vein), the hydrostatic pressure increases, leading to flow reversal with segmental PE. These events most commonly occur in patients with prolapse of the posterior mitral leaflet.

## Case presentation

A 74-year-old male presented to the emergency department with the chief complaint of progressive dyspnea on exertion and at rest for four weeks, associated with lower extremity edema and difficulty sleeping. The patient had been hospitalized three times due to symptoms perceived to be secondary to community-acquired pneumonia (CAP) or cryptogenic organizing pneumonia, requiring supplemental oxygen and antibiotics (third-generation cephalosporin, azithromycin, and ciprofloxacin) without improvement. His past workup included bronchoscopy, which did not assist in formulating a final diagnosis.

Pertinent medical history included chronic obstructive pulmonary disease on 2 L of supplemental oxygen, paroxysmal atrial fibrillation, mild/moderate MR, and essential hypertension.

**Figure 1:**
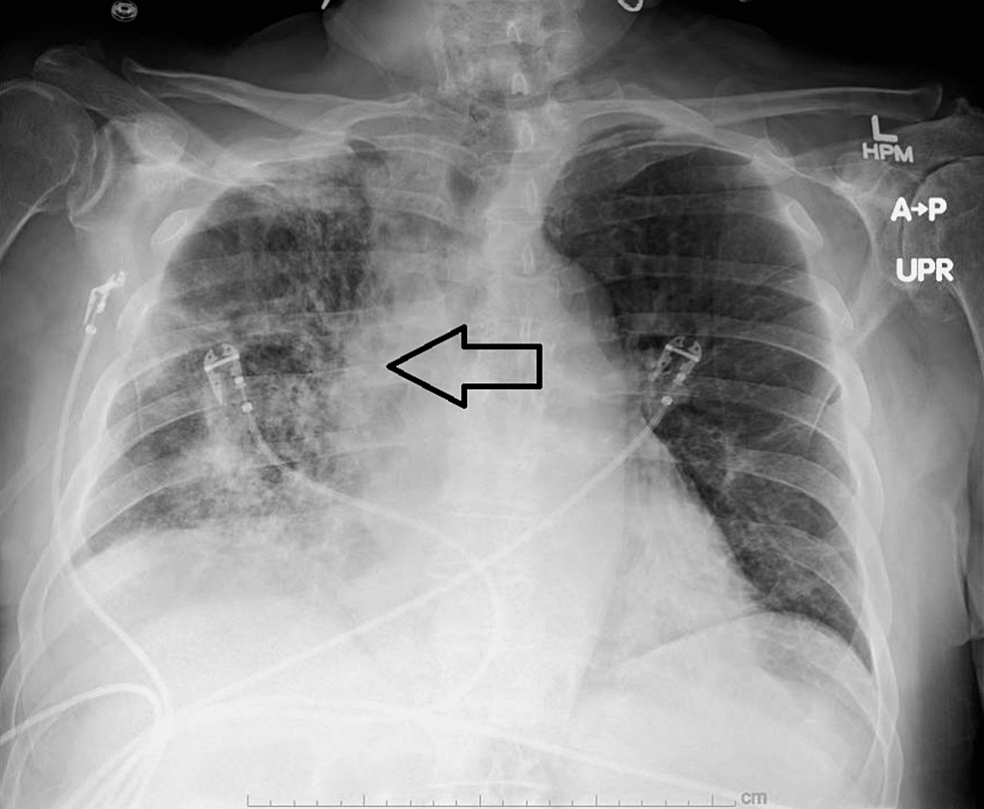
Our patient's admission AP chest radiograph showing unilateral, right-sided infiltrates AP: anteroposterior

**Figure 2:**
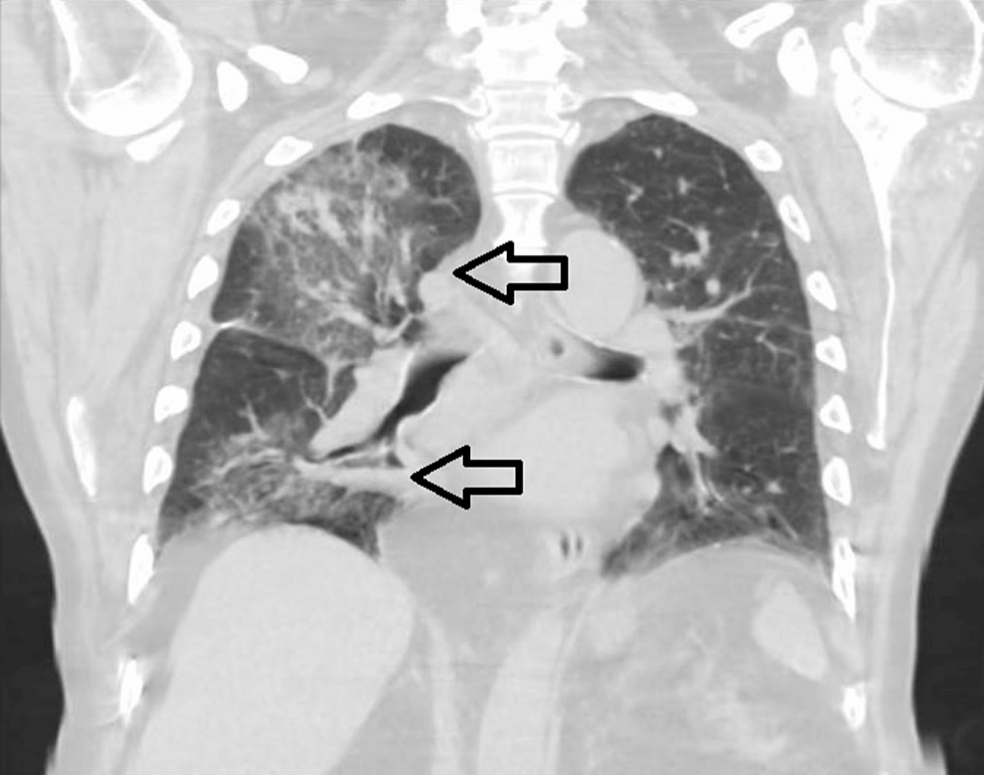
Computed tomography of the chest, coronal view, showing right-sided multi-lobar infiltrates

On presentation, he was afebrile at 36.6°C, heart rate 96 b.p.m, blood pressure 122/62 mmHg, and respiratory rate (RR) 28 breaths/min with an oxygen saturation of 89%, which improved to 95% on 5 L/min supplemental oxygen. The physical exam revealed right lung coarse crackles, irregularly irregular rhythm, and a 2/6 holosystolic murmur best heard in the mitral valve (MV) area radiating to the left axilla. Pertinent laboratory testing included leukocytosis (leukocyte count 13,700/μL), anemia (hemoglobin 12.2 mg/dL), and elevated NT-proBNP (4513 pg/ml). Chest X-ray demonstrated right-sided middle and lower lung infiltrates (Figure 1), and computed tomography (CT) of the chest revealed extensive ground-glass opacities in the right lung with moderate pleural effusion (Figure 2).

**Figure 3:**
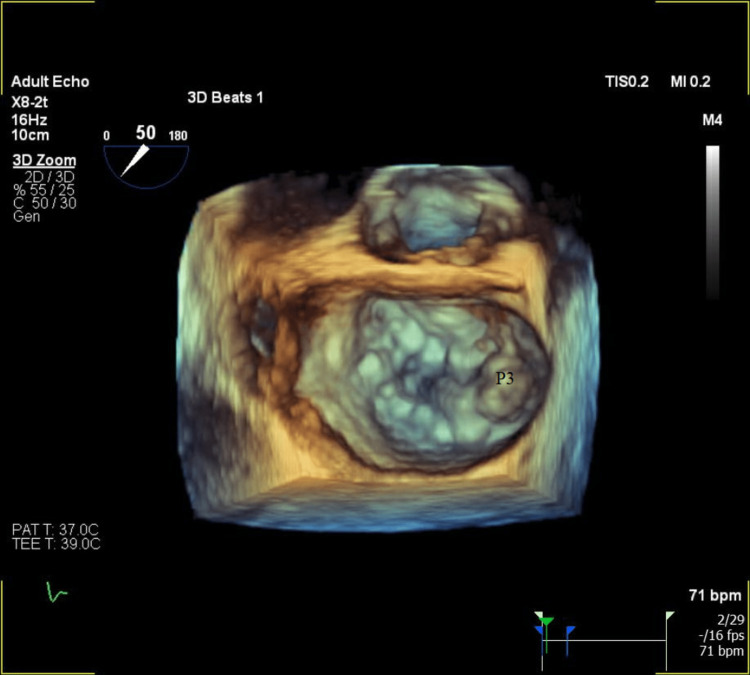
Preoperative transesophageal echocardiogram multiplanar 3D reconstruction of the mitral valve, en face view, showing the flail P3 segment during systole P3: flail P3 segment

The patient was admitted with a working diagnosis of acute hypoxic respiratory failure secondary to CAP. Unfortunately, his status continued to decline despite antibiotic therapy. Cardiogenic etiology for hypoxia and unilateral infiltrate was considered given his history of mild/moderate MR and elevated N-terminal pro-b-type natriuretic peptide (NT-proBNP). Further workup with a transthoracic echocardiogram (TTE) revealed a left ventricular ejection fraction of 54% and moderate/severe MR with a single jet, directed eccentrically and anteriorly. Thus, he was started on intravenous furosemide with minimal improvement in his symptoms. Subsequently, a three-dimensional transesophageal echocardiogram (TEE) was obtained, which demonstrated severe, anteriorly directed MR (Video 1) due to a flail posterior leaflet (P3 segment) with torn cords (Figure 3, Video 2). Measurements included an effective regurgitant orifice area of 1.1 cm^2^ and systolic flow reversal of the right upper pulmonary vein.

**Video 1:** Preoperative transesophageal echocardiogram Doppler showing an eccentric regurgitant jet toward the right upper pulmonary vein This leads to an increase in the mean capillary pressure within the right lung, resulting in unilateral pulmonary edema.

**Video 2:** Preoperative transesophageal echocardiogram multiplanar 3D reconstruction of the mitral valve, en face view, showing flail P3 segment. This causes an eccentric regurgitant jet towards the right upper pulmonary vein.

Cardiothoracic surgery was consulted for surgical intervention or placement of a transcatheter mitral clip. Furthermore, left and right heart catheterization was done. Given the location of the flail and the extent of the prolapse, he was deemed not a good candidate for a mitral clip. Consequently, the patient was recommended to undergo surgical repair or replacement of the MV. Considering his comorbidities, frail respiratory status, and to minimize operative time, surgical MV replacement was recommended and pursued. Intraoperative TEE demonstrated a normally functioning well-seating bioprosthetic MV (33 mm Magna Ease tissue valve; (Edwards Lifesciences, Irvine, CA)) with no paravalvular leaks and baseline ventricular systolic function (Video 3).

**Video 3:** Intraoperative transesophageal echocardiogram showing well-seating and properly functioning bioprosthetic valve, without residual regurgitant flow.

**Figure 4:**
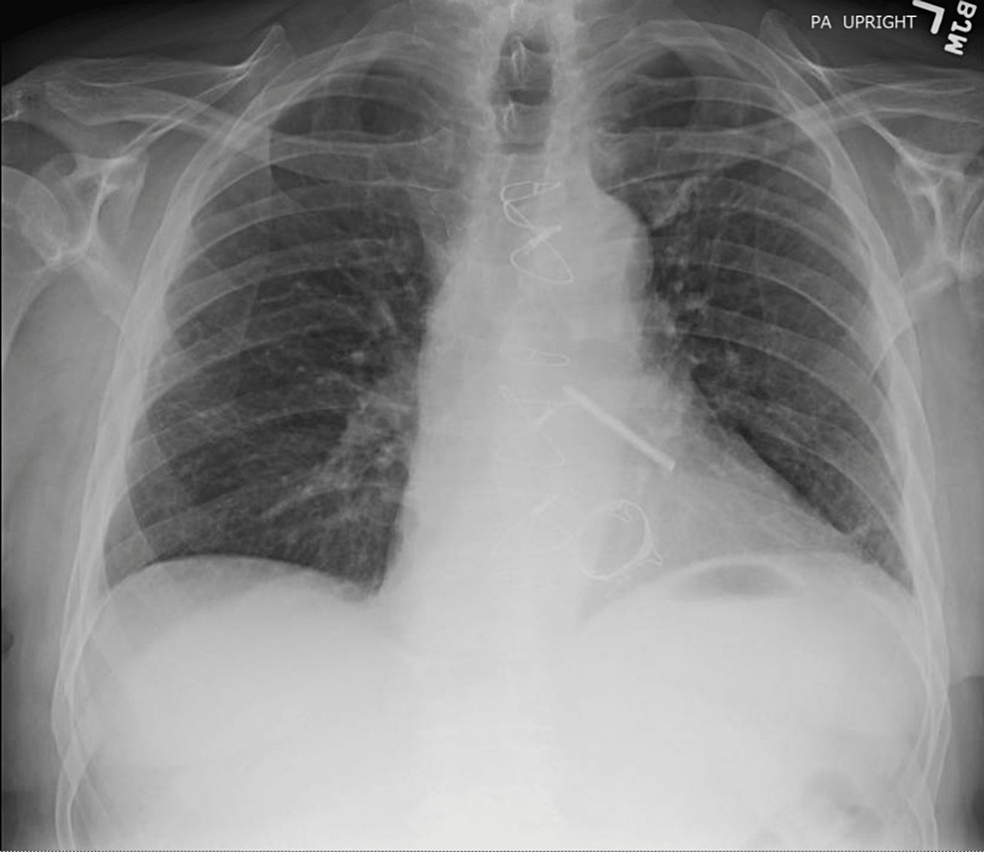
Our patient's PA chest radiograph, two weeks post-surgery, showing complete resolution of the infiltrates PA: posteroanterior

After valve replacement, there was a significant improvement in symptoms and exercise capacity. A repeat chest radiograph (Figure 4) a few weeks later showed clear lungs and a follow-up echocardiogram demonstrated resolution of the MR and normal left ventricular function.

## Discussion

This case illustrates that chronic MR should be considered a culprit in recurring unilateral pulmonary infiltrates. In these cases, early treatment for underlying valvular disease is associated with improved long-term outcomes [1]. This patient’s recurrent hospitalizations for unilateral infiltrates were incorrectly categorized as an infectious etiology (pneumonia), requiring multiple hospitalizations without resolution or significant improvement. Further testing for an alternative etiology revealed he had an unusual case of unilateral CPE.

Unilateral PE is a rare occurrence, and it represents around 2% of the cases of CPE [1]. In most cases, it affects the right lung [2]. A few hypotheses such as poorer lymphatic drainage of the right lung or physical limitation of blood flow toward the left lung due to cardiac enlargement in heart failure have been described in the literature. Nevertheless, the main cause is severe mitral regurgitation [3]. The pathophysiology behind it is mitral leaflet prolapse, which leads to an eccentric jet that is predominantly directed toward the upper right pulmonary vein. This increases the mean capillary pressure in the right lung, resulting in right-sided pulmonary edema.

Diagnosing unilateral CPE secondary to severe MR requires a high index of suspicion: thorough history taking, physical examination, and proper selection of workup, including electrocardiogram, imaging (chest X-ray, TTE, and TEE), and laboratory tests (NT-proBNP). Importantly, three-dimensional TEE has a greater diagnostic value compared to two-dimensional TEE, as it provides the en-face view of the MV, which simulates the surgeon's view through a left atriotomy and allows for better visualization of the valve anatomy. Once diagnosed, further tests are required to ensure the best treatment option is pursued.

Currently, MV surgery, either repair or replacement, is a class 1 recommendation for severe primary MR in symptomatic patients [4]. Importantly, MV repair is preferred to replacement when the anatomic cause is a degenerative disease and if a successful and durable repair is possible [5]. The success rate and durability of the repair are highly dependent on the cardiac surgeon. Moreover, MV repair is associated with lower operative mortality, better long-term survival, and fewer valve-related complications compared with valve replacement [6]. Conversely, MV replacement is preferred in patients with an extremely complex degenerative disease [7] and poor clinical status in order to limit surgical time. To date, there are no randomized studies comparing MV repair and replacement in patients with degenerative MR.

In patients with a high surgical risk, transcatheter edge-to-edge MV repair (MitraClip, Abbott, Abbott Park, Il), a more novel approach, is currently considered a class 2a recommendation [4]. The EVEREST II trial found that MitraClip has a comparable outcome at 1 and 5 years of follow-up in selected patients [8].

In our case, MV replacement was chosen as the best therapeutic intervention. The patient's age, compromised respiratory status, and increased surgical risk made him a poor candidate for MV repair, and his valve anatomy was not appropriate for MitraClip.

## Conclusions

Severe MR should be considered in the differential diagnosis of unilateral infiltrates. As it presents with unilateral lobar infiltrates on imaging studies, in the absence of a high index of suspicion, it can be easily misdiagnosed as pneumonia. As a result, this can lead to mistreatment and delay in patient care.

Finally, management should be tailored to the individual patient. The best therapeutic intervention should be adapted not only to multiple factors, such as the patient’s age, comorbidities, and surgical risk, but also the surgeon’s experience and success rate. It is currently known that mitral valve repair is the procedure of choice in most circumstances, but MV replacement should be considered in specific situations.
